# Systematic Comparison of OWAS, RULA, and REBA Based on a Literature Review

**DOI:** 10.3390/ijerph19010595

**Published:** 2022-01-05

**Authors:** Dohyung Kee

**Affiliations:** Department of Industrial Engineering, Keimyung University, Daegu 42601, Korea; dhkee@kmu.ac.kr; Tel.: +82-53-580-5319

**Keywords:** musculoskeletal disorders, observational technique, OWAS, RULA, REBA

## Abstract

This study aimed to systematically compare three representative observational methods for assessing musculoskeletal loadings and their association with musculoskeletal disorders (MSDs): Ovako Working Posture Analysis System (OWAS), Rapid Upper Limb Assessment (RULA), and Rapid Entire Body Assessment (REBA). The comparison was based on a literature review without time limitations and was conducted on various factors related to observational methods. The comparisons showed that although it has a significant limitation of comprising only two classifications for the leg postures, (1) the RULA is the most frequently used method among the three techniques; (2) many studies adopted the RULA even in evaluation of unstable lower limb postures; (3) the RULA assessed postural loads as higher risk levels in most studies reviewed in this research; (4) the intra- and inter-reliabilities for the RULA were not low; and (5) the risk levels assessed by the RULA were more significantly associated with postural load criteria such as discomfort, MHTs and % capable at the trunk, and MSDs.

## 1. Introduction

Work-related musculoskeletal disorders (WMSDs) are one of the most frequent occupational disabilities in the industry. WMSDs accounted for approximately 67% of occupational injuries and illness in Korea in 2019 and 29–35% in the USA in 1992–2010 [1,2]. WMSDs cost approximately 40% of the global compensations for occupational and work-related accidents and diseases [3]. Work-related musculoskeletal load due to awkward or static postures, excessive amount of force, repetitive effort, etc., is known to be a strong risk factor for developing WMSDs [4]. For managing and preventing WMSDs, it is critical to assess exposure to risk factors of WMSDs and to implement intervention programs that reduce the load to acceptable levels for workers [5,6,7].

Therefore, ergonomists and practitioners use various tools for quantifying exposure levels. Of the varying assessment methods, observational techniques have been used more frequently. While the use of direct measurement approaches, including motion capture/measurement, electronic goniometer, and push/pull force sensors, has minimally increased, the use of observational methods, such as Rapid Upper Limb Assessment (RULA) [8], Rapid Entire Body Assessment (REBA) [9] and Ovako Working Posture Analysis System (OWAS) [10], by US ergonomists has significantly increased in 2017, compared to that in 2005 [11]. The observational techniques are inexpensive, easy to use, flexible, and do not interfere with workers’ tasks or the jobs being performed [5,12,13,14,15,16].

Although many observational methods have been developed and applied for assessing musculoskeletal disorders (MSDs)-related risk factors, the OWAS, RULA, and REBA have been most frequently applied in industries for assessing the load of the whole body [11,17]. The OWAS technique was developed by Ovako Oy, a Finnish steel company, and identifies four work postures for the back, three postures for the arms, seven postures for the lower limbs, and three categories of the weight of loads handled or amount of force used [10]. The RULA was proposed to provide a quick assessment of the load on the musculoskeletal system due to the postures of the neck, trunk, and upper limbs, muscle function, and external loads exerted [8]. The REBA technique is a postural analysis system that is sensitive to musculoskeletal risks in a variety of tasks, especially in the assessment of the working postures found in health care and other service industries [9]. While the OWAS and REBA classify the joint motions of the whole body into some groups, the RULA focused on the classification of the upper body including the trunk.

The three techniques have been cited in relevant literature approximately 1670–3680 times, which resulted in far more citations than any other observational method [6,14,18]. Joshi and Deshpande [19] reported that of the 18 observational techniques dealt with in their study, the REBA (69%) was compared most frequently, followed by the RULA (64%), Strain Index (36%), and OWAS (33%), based on 39 comparative studies.

Many previous studies have summarized and compared the three techniques, based on their general characteristics including correspondence with a valid reference, scales for posture classification, main functions, association with MSDs, intra- and inter-observer repeatability/reliability, potential users, and exposure factors assessed such as postures or body regions, load/force, and movement frequency [5,15,16,17,19,20]. Some studies compared the three methods based on their assessment results [12,20,21,22,23,24,25,26,27], and the relationship between the epidemiological data or subjective symptoms of MSDs and assessment results or scores [27,28,29,30,31,32,33].

The above studies have compared the three techniques from a few perspectives that were different from one another. Consequently, these existing studies make it difficult to draw a comprehensive decision on which method is better or more suitable for a situation. Therefore, this study aimed to systematically compare the three observational techniques of the OWAS, RULA, and REBA based on a literature review, which included almost all perspectives mentioned above.

## 2. Materials and Methods

The comparison of the three observational techniques was mainly based on a literature survey. The author of this study has conducted several relevant studies, and already had much of the literature considered in this study. Other relevant literature was supplemented based on the reference lists of these articles and via a search of the electronic databases such as ScienceDirect and Scopus without time limitations using the following keywords: “OWAS,” “RULA” and “REBA.” Of the 190 papers/articles searched, 109 studies that were not related to this study, included a method or did not deal with any of the comparison factors used in this study were discarded. Whether a study was included in this study was judged based on its title and abstract. Only peer-reviewed journal papers that dealt with more than two methods or at least a comparison factor, and were written in English were considered. However, the author excluded those studies that were presented at conferences or found in edited-book chapters. Of the remaining 81 studies, 47 studies were performed or owned by the author. Thirty-four studies were newly selected for this study through the search process. A total of 81 studies were used in the subsequent comparisons.

Although there is no generally accepted standard way to systematically compare or evaluate observational techniques [16], this study compared the three techniques based on the general characteristics, applied fields, risk levels, agreements and correlations between methods, intra- and inter-rater reliability, and validations. The perspectives mentioned above have been used in existing studies that compared or evaluated ergonomics assessment tools. The validations were investigated by analyzing correspondence with valid references and associations with MSDs [16].

The OWAS and RULA classify postural loads for the urgency of corrective actions into four action categories and action levels, respectively, the meanings of which are similar. The REBA groups postural loads into five action levels, which have slightly different interpretations from the action categories/levels of the OWAS/RULA. To facilitate an effective comparison, the five REBA action levels were regrouped into four categories by considering the meanings of the action categories/levels in these three techniques. Therefore, the four new REBA action levels were as follows: action level 1 (originally action level of 0), 2 (originally action levels of 1 and 2), 3 (originally action level of 3), and 4 (originally action level of 4). This regrouping is identical with that by Kee and Karwowski [24].

In case agreement rates and correlations among the three methods, which will be employed as comparison factors in the following, were not presented in the original corresponding studies, the values were calculated with using the relevant results provided in the original studies or experimental data by the author, if possible.

## 3. Results

### 3.1. General Characteristics

Kee [23] summarized the general characteristics of the three methods, based on previous studies [5,16,17,19], the results of which are shown in Table 1. While the OWAS assesses only the loadings of posture and force/external load, the RULA and REBA evaluate the exposure to posture and force/external load as well as repeated and static posture effects. The REBA has two additional assessment factors of coupling and dynamic loading effects, compared to the RULA. The OWAS does not categorize the left and right upper extremities during assessment, but the RULA and REBA evaluate only one side at a time, which is considered to be under greater stress. If it is difficult to decide which side is more loaded, both sides are assessed. The OWAS assesses postural loads based on the time sampling, while the REBA selects and estimates the most common or prolonged, or loaded postures [16]. However, it is general for the OWAS and RULA to observe the most common or prolonged, or loaded postures as in the REBA. The three observational methods comprise of four or five action categories or levels for classifying the risk category [8,9,10]. The three techniques do not consider the effects of recovery, duration, vibration, environmental conditions, and psychosocial and individual factors, which have been known to affect the occurrences of MSDs [12,16,34,35]. The relative strengths and limitations of the three methods are briefly summarized in Table 1, based on Takala et al.’s study [16].

### 3.2. Application Fields

Among 166 OWAS-applied studies published between 1977 and 2017, Gómez-Galán et al. [14] identified a total of 12 work environments assessed in the studies. The OWAS was the most frequently applied technique in manufacturing industries with a total of 34 published articles, followed by healthcare and social assistance activities (22), information and communication (19), agriculture, livestock, forestry and fishing (19), transportation and storage (10), and construction (9).

Gómez-Galán et al. [18] stated that based on 226 RULA-relevant publications between 1993 and 2019, the manufacturing industries with 74 publications overwhelmingly ranked first in the applications of the RULA, followed by human health and social activities (38); agriculture, forestry, and fishing (18); transportation and storage, administrative and support service activities, and education (12); and information and communication (9).

Hita-Gutiérrez et al. [6] investigated the application fields of the REBA concerning 91 studies between 2002 and 2019. The number of applications was ranked (from most to least) as follows: (1) manufacturing industries (22), (2) agriculture, forestry, and fishing (20), (3) human health and social activities (15), (4) transportation and storage (4), and construction (4).

### 3.3. Risk Levels by Methods

The 44 studies that dealt with assessments for postural loads using two or more of the three methods are summarized in Table 2. The summary included the author(s) of the studies, application fields, sample size, and rank order for risk levels evaluated by the three methods. The three techniques have been applied to various fields such as aerospace, agriculture, bicycle repairing, cattle slaughter, construction, experimental environment, food, forestry, hospital, kitchen, laboratory, lifting tasks, manufacturing, potter and sculptor, sawmill, service industries, tailors, typist, and welders. The sample sizes for the applications varied from two working postures [36] to 4251 postures [37]. For effective comparison, some studies regrouped postural loads into three or four risk levels with consideration of the meanings of risk categories for the techniques adopted. The number of risk levels reclassified is specified in the column of ‘Remarks’ in Table 2. Cremasco et al. [38] adopted the normalized values based on the minimum–maximum transformation for comparing the differences between the REBA and RULA scores.

Nine of the 10 studies dealing with the OWAS and RULA applications revealed that the RULA assessed postural loads as higher risk levels than the OWAS. A study by Mukhopadhyay et al. [39] showed that the two techniques equally evaluated nine activities performed in bicycle repairing units as action level 4.

Of the 13 studies in which the OWAS and REBA were simultaneously applied, 10 studies except those by Kee et al. [25], Isler et al. [37], and Mukhopadhyay et al. [39] concluded that the REBA-rated postural loads were more stressful than the OWAS-rated postural loads. According to Kee et al. [25], the OWAS evaluated postural loads for 72 experimental postures as more stressful than the REBA, but the differences were not statistically significant (Wilcoxon signed-rank test, *p* > 0.20). Isler et al. [37] estimated postural loads for 4237 and 4251 postures in the clothing sector using the OWAS and REBA, respectively, which resulted in no significant differences between the postural loads by the OWAS and REBA (Paired *t*-test, *p* > 0.10). Mukhopadhyay et al. [39] reported that the OWAS and REBA assessed nine activities in bicycle repairing with the same action category or level 4.

Of the 36 studies that adopted the RULA and REBA as ergonomic risk assessment tools, 30 studies demonstrated that the RULA estimated postural loads for the selected postures more stressfully than the REBA. Meanwhile, of the six studies in which the RULA did not evaluate postural loads as more stressful than the REBA, two studies by Mukhopadhyay et al. [39], and Pal and Dhara [36] exhibited that the RULA and REBA identically evaluated the postures selected in the bicycle repairing and uprooting job of rice cultivation, respectively. According to Balaji and Alphin [40], and Bhatia and Singla [41], there were no significant differences between assessments by the RULA and REBA. Kulkarni and Devalkar [42] claimed that while the RULA evaluated five activities such as granite cutting, brickwork, shuttering, plastering, and material transportation in construction as action level 3 or 4, the REBA evaluated them as action level 4. Sain and Meena [43] indicated that while the RULA rated 154 postures sampled from four tasks including spading, mold filling, mold evacuating, and brick carrying in clay brick kiln work as action level 3 on average, the REBA evaluated the postures for spading and mold filling as action level 3, and for mold evacuating and brick carrying as action level 4.

The above showed that the RULA generally rated postural loads as the highest risk levels, the REBA as the second, and the OWAS as the third.

### 3.4. Agreement Rates between Methods

Some studies presented the results following assessment by two or three methods, but did not explicitly provide the agreement rates between the methods in the original articles. The agreement rates by Cremasco et al. [38], Kulkarni and Devalkar [42], Gallo and Mazzetto [47], Garcia et al. [48], Noh and Roh [49], Sahu et al. [52], and Paini et al. [67] were calculated using the assessment results presented in the original studies by the author of this study, and those by Kee et al. [25] were obtained using the experimental data. The agreements among the three methods varied depending on the studies (Table 3). While the values between the RULA and REBA by Chiasson et al. [12], Pal and Dhara [36], Cremasco et al. [38], Jones and Kumar [46], Kulkarni and Devalkar [42], Sahu et al. [52], and Qureshi and Solomon [72] were not less than 60%, the rates in other studies were lower (≤50.0%). The agreement between the OWAS and RULA by Garcia et al. [48] was null (ĸ = 0). In the study, the OWAS estimated the prevalence of risk of upper limb MSDs as ‘low’ or ‘medium,’ while the RULA evaluated it as ‘high’ or ‘extremely high.’ The agreement rates between the OWAS and RULA, and between the OWAS and REBA for most studies except two studies [24,25] were ≤50%. The mean agreement between the RULA and REBA was higher than those between the OWAS and RULA and those between the OWAS and REBA.

### 3.5. Correlations between Methods

A few studies provided the correlation coefficients between the three methods (Table 4). The coefficients by Chiasson et al. [12] were assumed to be based on the action level or category of the OWAS, RULA, and REBA. Kee [23] and Kee et al. [25] used the OWAS action category, RULA grand score, and REBA score for the correlation analyses. Although there were no correlation coefficients between the three techniques in the original articles by Kee and Karwowski [24], Kee et al. [25], and Kee [30], the coefficients were based on the author’s calculation using the source or experimental data. The highest and most significant correlation was between the OWAS and REBA (0.785) by Kee et al. [25], followed by the correlation between the RULA and REBA (0.691 and 0.67 by Kee et al. [25], and Chiasson et al. [12], respectively). Other coefficients except two (0.511 and 0.562 for the OWAS and RULA by Kee and Karwowski [24] and Kee [30], respectively) were <0.5. The correlation coefficients between the OWAS and REBA, and the RULA and REBA were larger than that between the OWAS and RULA.

### 3.6. Inter- and Intra-Rater Reliability

Karhu et al. [10] and de Bruijin et al. [73] presented the intra- and inter-rater reliabilities of the OWAS, using 36,240 observations of 52 tasks in their original study on the OWAS, and 45 slides showing nurses in different working postures, respectively. The intra-rater reliabilities by Karhu et al. [10] and de Bruijin et al. [73] ranged from 70 to 100% and from 83 to 100%, respectively (Table 5). The intra-rater reliabilities determined on a 4 week-interval by de Bruijin et al. [73] ranged from 88 to 97% (mean: 92%), and those for a 3.5 month-interval ranged from 83 to 100% (mean: 90%) depending upon the body parts. Five studies by Karhu et al. [10], de Bruijin et al. [73], Kivi and Mattila [74], Mattila et al. [75], and Lins et al. [76] provided the inter-rater reliabilities for the OWAS, the agreement values of which ranged from 23% to 100% depending on the raters and body parts. Lins et al. [76] presented the ĸ values together with the agreement rates according to the body parts assessed, the values of which corresponded to ‘very good.’ They also found no significant differences between raters with and without prior training in physical therapy.

McAtamney and Corlett [8] claimed in the original study dealing with the RULA that there was a high consistency of scoring among 120 raters. Dockrell et al. [77] reported the intra- and inter-rater reliabilities for six raters (three physiotherapists and three undergraduate physiotherapy students), based on the 24 children’s computing postures. The interclass correlation coefficients (ICCs) of the intra-rater reliability were 0.27–0.86 for the action levels and 0.47–0.84 for the grand scores depending upon the raters. The ICCs of the inter-rater reliability were 0.54–0.72 for the action levels and 0.50–0.77 for the grand scores. In addition, the intra-rater reliability was generally higher for the older children than for the younger children, especially when the physiotherapists assessed the postures. Laeser et al. [78] found that the inter-rater reliability for keyboarding and mousing tasks of 58 students in the sixth and eighth grades using Kendall’s coefficient of concordance was statistically significant (Kendall’s W = 0.773) and that the median score of a team of independent observers for four subjects’ videotaped postures was significantly correlated with the lead investigator’s ratings (the first author of the study) (Pearson’s r = 0.96, *p* < 0.05). Breen et al. [79] and Oates et al. [80] showed that the inter-rater reliability of the RULA was 94.6% and Ebel r = 0.73 based on the observations in children using computers, respectively.

Lamarão et al. [81] and Schwartz et al. [82] stated that the intra-rater reliabilities for the REBA was ‘poor’ to ‘moderate’ (linear weighted ĸ values: 0.104–0.516; agreement rates: 15.09–69.81%) according to the body regions assessed, and the ICC of 0.925 for the raw scores, respectively. Four studies by Hignett and McAtamney [9], Lamarão et al. [81], Schwartz et al. [82], and Jantowitz et al. [83] exhibited that the inter-rater observer reliability was 62–85%, ĸ value 0.126–0.454 (‘poor’ to ‘moderate’) (5.66–66.03%), the Fleiss ĸ value of 0.54 (‘moderate’), and ĸ value 0.20–0.66 except for the left wrist (‘fair’ to ‘good’), respectively.

Widyanti [84] examined the inter-rater reliabilities of the OWAS, RULA and REBA, based on the % agreement and ĸ value among 50 new raters. The mean inter-rater reliabilities of the OWAS, RULA and REBA were 57.07% (ĸ value 0.39), 58.25% (ĸ value 0.20) and 50.14% (ĸ value 0.26), respectively. There were no significant differences in % agreements and ĸ values among the three methods. In summary, the inter- and intra-rater reliabilities of the OWAS were the highest, those of the RULA ranked second, and those of the REBA ranked third.

### 3.7. Validation of the Three Methods

Previous studies by Kayis and Kothiyal [85], Olendorf and Drury [86], and Hellig et al. [87] argued that the assessments by the OWAS were associated with the subjective load criteria such as Borg scale [88] and perceived exertion scores (Table 6). Kayis and Kothiyal [85] and Hellig et al. [87,89] indicated that the OWAS action levels were in agreement or correlated with the biomechanical measures including the L5/S1 compressive forces and corresponding muscle activities. On the contrary, van der Beek et al. [90] asserted that the ranks for distinct scaffolding tasks determined by the OWAS were different from those determined by the revised NIOSH (National Institute for Occupational Safety and Health) lifting equation [91], lifting guidelines for the Dutch construction industry (Arbouw method) [92], and rapid appraisal of the NIOSH lifting equation (practitioner’s method).

McAtamney and Corlett [8] suggested that the neck and lower arm scores, and scores A and B by the RULA were significantly related to the self-reported pain, ache, or discomfort in the neck and lower arm, and the relevant functional unit regions, respectively (*p* < 0.01). Several studies showed that the RULA action levels, grand scores, and body part scores were associated with electromyography signal (root mean square amplitude), discomfort, job attitude scores, and maximum holding time (MHT) [23,25,79,93]. Massaccesi et al. [31] indicated that the trunk and neck scores by the RULA were significantly associated with self-reported pains, aches, or discomforts in the trunk and neck regions. The self-reported pains, aches, or discomforts in the above two studies were measured using the Body Discomfort Chart by Corlett and Bishop [94]. Shuval and Donchin [33], and Yazdanirad et al. [27] reported that the assessment results or scores by the RULA were associated with the prevalence of the upper extremity symptoms evaluated using the Nordic Musculoskeletal Questionnaires [95].

Rathore et al. [32] pointed out, based on the multivariate logistic regression models, that the REBA scores A and B and REBA scores were significantly correlated with musculoskeletal symptoms for the different body regions. The data pertaining to the subjective MSD symptoms were collected using the Cornell Musculoskeletal Discomfort Questionnaire [96].

Domingo et al. [29], and Vahdatpour and Sayed-Marramazani [68] dealt with the association of subjective MSD symptoms with postural loads assessed by two of the three methods (RULA and REBA, and OWAS and RULA, respectively). The former showed the inconsistency between the results of the subjective symptoms and the assessments by the RULA and REBA (Spearman rank-order correlation coefficient: 0.1 for RULA and 0.46 for REBA). The latter asserted based on the Spearman correlation coefficients that although the OWAS action level had no significant association with the incidence of MSDs, the level of risk based on the RULA had a direct relationship with the incidence of MSDs in the neck, upper back and knees (*p* < 0.05).

Some studies have compared and validated the three techniques simultaneously. Choi et al. [21], and Kong et al. [26] estimated 196 working postures of agricultural tasks with the Agricultural Upper Limb Assessment (AULA), Agricultural Lower Limb Assessment (ALLA), OWAS, RULA, and REBA, and compared the assessment results with the subjective evaluations using a 10-point scale by 16 ergonomic experts. The studies revealed that based on the quadratic weighted ĸ values, the RULA was in ‘moderate’ and ‘good’ agreements depending on the studies (0.599 and 0.627, respectively), whereas the OWAS and REBA were in ‘moderate’ agreement (0.538 [21] and 0.501 [26] for OWAS, and 0.578 [21] and 0.490 [26] for REBA) with the expert evaluations.

Kee [23,30] and Kee et al. [25] compared the three techniques based on perceived discomfort, MHT, % capable at the shoulder and trunk, and association with MSDs using correlation analysis, chi-squared test, and logistic regression analysis. The studies revealed that the RULA grand score and REBA score were significantly correlated with discomfort, MHT, and % capable at the trunk [23,25], and that the RULA grand score and action level, and REBA action level were significantly associated with MSDs [30].The OWAS action category was significantly associated with % capable at the trunk, but the correlation coefficient was lower. However, the OWAS action category was not significantly correlated with discomfort and MHT [23,25], and the OWAS action category and REBA score were not associated with MSDs [30]. The OWAS action category, RULA grand score and REBA score were not significantly correlated with % capable at the shoulder [23]. The research by Kee [30] exhibited that the percentage concordances for the RULA grand score and action level (52.4% and 44.8%, respectively) were significantly higher than those for the REBA action level (22.1%) in the logistic regression analyses.

Widyanti [84] presented the validities for the OWAS, RULA and REBA, based on the correlation between new 50 raters’ ratings and an ergonomics expert’s ratings. Here, the expert’s rating was used as the gold standard. The study claimed that there were no significant differences between ratings by the new raters and the ergonomics expert for the OWAS, RULA and REBA (Wilcoxon signed-rank test, *p* > 0.05), and that there were significant correlations between the ratings of the new raters and those of the expert for the OWAS (r = 0.802, *p* < 0.01), RULA (r = 0.799, *p* < 0.01), and REBA (r = 0.790, *p* < 0.01).

The number of validation studies for the OWAS and RULA (13 and 14, respectively) was higher than that for the REBA (8). The validations are summarized as follows: (1) while nine studies on the OWAS showed affirmative results in viewpoints of correspondence with valid references and association with MSDs [21,26,28,84,85,86,87,88,89], four studies reported negative results [25,30,68,90]; (2) 13 studies (except a study [29]) for the RULA were affirmative; and (3) seven studies (except one [29]) for REBA were also affirmative.

## 4. Discussion

This literature review systematically compared three observational techniques for assessing postural loads and/or whether the assessment results were associated with MSDs, with respect to varying viewpoints including the general characteristics, applied fields, risk levels, agreements and correlations between methods, intra- and inter-rater reliability, and validations. The findings indicated that although it focused on the classification of the upper limb postures rather than the whole-body postures, the RULA may have some advantages in estimating postural loads and the association with MSDs, application fields, intra- and inter-rater reliability, and validations among the three methods. This agrees with the findings of previous studies suggesting that the RULA gives more sensitive assessment results compared to the OWAS and REBA [58,69]. Consequently, the RULA is a more precautionary method to protect the health of the workers or operators during work [38].

Although the three methods have been applied in various fields for assessing postural loads, they have been most frequently adopted in the manufacturing industry, followed by in health and social activities, agriculture, forestry and fishing, information and communication, and transportation and storage [6,14,18]. Many studies used the RULA for assessing workloads in the administrative and support service activities, and education involving considerable sedentary work, due to its characteristics, focusing on the upper limb postures [18]. Shah et al. [97] used the RULA for analyzing sitting postures and the REBA for assessing standing postures, following the methods’ characteristics. However, the RULA has often been employed for rating postural loads even in the agriculture, forestry and fishing, mining and quarry, construction, and shipbuilding industries as well as general hospitals, which require frequent unstable or awkward lower limb postures such as squatting and kneeling [18,98].

Many studies have evaluated postural loads in industrial settings and experimental environments using two or three techniques of the OWAS, RULA, and REBA. Of these, studies using the RULA and REBA were the most common (36 studies), followed by those using the OWAS and REBA (13 studies) and the RULA and OWAS (10 studies) (Table 2). The literature review revealed that the RULA assessed postural loads for corresponding postures as more stressful or identical, compared to the OWAS and REBA in 10 of 10 studies (100.0%) and 34 of 36 studies (94.4%), respectively, and that the REBA estimated postural loads as more stressful or the same, compared to the OWAS in 12 of 13 studies (92.3%). In summary, 36 of 38 studies exhibited that the RULA rated postural loads in various fields more stressfully or the same, compared to the OWAS and REBA. Of four studies that the RULA evaluated musculoskeletal loads as the same risk level as the OWAS or REBA, two studies by Balaji and Alphin [40], and Bhatia and Singla [41] showed that there were no statistically significant differences between assessments by the RULA and REBA. A study by Pal and Dhara [36] had too small sample sizes of two postures to draw a general conclusion on the order of postural loads evaluated by the two or three techniques. Mukhopadhyay et al. [39] found that the three techniques equally evaluated nine activities performed in bicycle repairing units as action level 4. This may be because the postural loads for the nine bicycle repairing activities were sufficiently high to reach or exceed those of action category/level 4. The above implies that the RULA tends to generally evaluate postural loads as the most stressful, followed by the REBA and the OWAS.

The agreement rates between the RULA and REBA were found more frequently than those between the OWAS and RULA, and those between the OWAS and REBA, and were higher on average, followed by the rates between the OWAS and REBA, and those between the OWAS and RULA (Table 3). This trend may be partly attributed to the rank of the risk levels for the three methods (RULA > REBA > OWAS). The agreements varied widely ranging from null (ĸ = 0) to 100%, which means that the risk assessment results by the three methods do not agree or correlate well.

The correlation coefficients among the three techniques varied from 0.415 to 0.785 according to the studies (Table 4); however, there was no established trend among the coefficients. Most studies showed low correlation coefficients of <0.5, which implies that the three methods yielded assessment results which were different from one another. The reasons for the low agreements or correlations may be the differences in the ability to assess musculoskeletal loads and the risk levels such as the action level and category between the three methods, and the different weights assigned to risk factors when calculating a score (scores A, B, C, and D and grand score in the RULA; scores A, B, and C and REBA score in the REBA) [99,100].

It is challenging to directly compare the intra- and inter-rater reliabilities for the three techniques (Table 5), because different measures such as agreement rate (%), ICC, Kendall’s W, Ebel r, and ĸ values were adopted according to various studies. The two ratings for evaluating the intra-rater reliability should be conducted at a sufficiently long time interval (2–3 weeks) to prevent recall bias [101]. However, Karhu et al. [10] tested the intra-rater reliability in the morning and afternoon of the same day, which might have resulted in low reliability. Based on this study’s review (Table 5), it can be concluded that the intra- and inter-rater reliabilities for the OWAS were the highest, followed by those for the RULA and REBA, in that order. This may be attributed to the degree of complexity of the three methods. In other words, the intra- and inter-rater reliabilities for the most simple and easy-to-use method, namely OWAS, were the highest. The result was in agreement with those of the study by Takala et al. [16]. Except for the three studies by Hignett and McAtamney [9], McAtamney and Corlett [8] and Widyanti [84], the number of raters who participated in the studies was very small (1–9 raters). It is known that discrepancies among raters frequently occur when a body segment is at a border between two ranges of the evaluation factors such as body posture, external load, and dynamic activities [8].

The validity of an observational technique can be evaluated by comparing it with a more valid method (concurrent validity, which assesses the correspondence of a method with more valid ones) or by estimating the association of its assessment results with MSDs (predictive validity, which assesses the association of risk estimates by a method with MSDs) [16]. This study summarized the validations by several studies (Table 6). While 12 of the 21 studies (57.1%) were classified into concurrent validity [21,23,25,26,79,84,85,86,87,89,90,93], 10 studies were included under predictive validity [8,27,28,29,30,31,32,33,68,93]. Of the validation studies, the study by Fountain [93] was corresponded to both concurrent validity and predictive validity. The concurrent validities were performed by comparing the assessment results of the three methods with subjective measures of Borg scale, exertion, discomfort, MHT, job attitude scores, ergonomic experts’ evaluations, and objective criteria including L5/S1 compressive forces, outputs of revised NIOSH lifting equation, and muscle activity. Ten predictive validity studies were based on the prevalence of MSD symptoms and association with MSDs. Nine predictive studies obtained the data regarding MSD symptoms using questionnaires [8,27,28,29,31,32,33,68,93], while a study by Kee [30] acquired the real MSD cases diagnosed by medical doctors from the industrial sites of automobile and automotive parts manufacturing, and construction. The nine predictive validities except Kee [30] investigated the association of assessment results by one of the three techniques with the subjective MSD symptoms, which makes comparisons for the three methods impossible. Kee [30] examined the association with real MSD cases with the risk levels evaluated using the three methods and concluded that the RULA could predict the association with MSDs more accurately than the OWAS and REBA. The RULA action level was more significantly associated with MSDs than the REBA action level (*p* < 0.01 and *p* < 0.05 in the chi-squared test, respectively). The former showed higher percentage concordance than the latter in the logistic regression analysis (44.8% and 22.1%, respectively).

The review results should be interpreted with caution, because (1) some studies had extremely small sample sizes or number of raters (sample sizes for Pal and Dhara [36], Cremasco et al. [38], Noh and Roh [49], Boulila et al. [60], Dev et al. [61], Paini et al. [67], and Kamath et al. [71] in Table 2 were <10), (2) the agreements and correlations showed large standard deviations (Table 3 and Table 4), and (3) this study is mainly based on the literature by various researchers with different experiences and fields of knowledge. These may generate bias in their results. For obtaining more reliable assessment results, and intra- and inter-reliabilities, further research with statistically sufficient sample sizes is required.

It should be noted that although this study systematically compared three representative observational methods using categorized or itemized characteristics frequently found in relevant studies, it is not easy to directly compare observational techniques and to draw general conclusions due to their own strengths and limitations, and differences between such techniques. The differences may be attributed to the characteristics of methods, including the applicable fields/settings/populations, risk factors assessed (e.g., posture, repetition, intensity, force exerted, vibration, coupling, and temperature), body parts (e.g., whole body, upper limb, and arm/hand), application procedures, observation strategy, decision rules, evaluation outcomes (e.g., quantitative risk score and qualitative job analysis), etc. Further studies to integrate and interpret the itemized comparison results may be required to obtain more general conclusions.

## 5. Conclusions

For overcoming shortcomings of existing studies dealing with one or two methods of the three techniques or comparing the methods in terms of a few viewpoints, this study systematically compared the OWAS, RULA, and REBA methods based on several viewpoints related to the observational methods, such as the general characteristics, applied fields, risk levels, correlations and agreements between methods, intra- and inter-rater reliability, and validations. The results showed that (1) the RULA is the most frequently used method among the three techniques in the US [11]; (2) many studies adopted the RULA with just two postural codes for the legs to assess postural loads where unstable or awkward lower limb postures, such as squatting and kneeling, occurred frequently; (3) in most studies reviewed in this research, the RULA assessed postural loads as higher risk levels, compared to the OWAS and REBA; (4) the intra- and inter-reliabilities for the RULA were not low (or moderate); and (5) the risk levels assessed by the RULA were more significantly associated with MSDs than with that by the OWAS and REBA.

It may be asserted, based on the above, that the RULA is better suited for assessing postural loads and the association with MSDs. This may be backed by the statement that in the safety field, it may be more desirable to estimate certain postures or tasks in industries as more stressful in preventing WMSDs, rather than to assess them as less stressful [23].

Although the RULA has some advantages stated above, it should be noted that there may be sensitive and insensitive zones according to the evaluation factors and scores (A and B) in the RULA [102]. The percentage concordance for the RULA was not high (44.8%) based on the logistic regression analysis. Further study for developing a new observational technique with less insensitive zones and more significant association with MSDs will be needed.

## Figures and Tables

**Table 1:** General characteristics of OWAS, RULA and REBA.

	Assessment Factors						Observation Strategy	Body Side Assessed	Risk Category	Strengths	Limitations
	Posture	Force/External Load	Motion Repetition	Static Posture	Dynamic Loading **	Coupling					
OWAS	Back, arms, legs	3 categories	X *	X	X	X	Time sampling	Not specified	4 action categories	Most rapid and easy to use Detailed leg posture classificaion	Postures of neck, elbow, and wrist, repetition, coupling, and static posture not included
RULA	Upper arms, lower arms, wrist, neck, trunk, leg	4 categories	O *	O	X	X	No detailed rules	Right or left side	4 action levels	Rapid and easy to assess	Focused on upper limb posture Coupling not included Necessity to decide which side to observe
REBA	Upper arms, lower arms, wrist, neck, trunk, leg	3 categories (+1 adjusting factor)	O	O	O	O	Most common/prolonged/loaded postures	Right or left side	5 action levels	Rapid and easy to assess	Necessity to decide which side to observe

* O: included; X: not included; ** Dynamic loading means rapid large changes in posture or an unstable base.

**Table 2:** Risk levels by studies.

Study	Application Fields	Sample Size	Rank Order for Risk Levels	Remarks
Chiasson et al. [12]	Aerospace, food, appliances, musical instruments, tree nurseries, plastics, and composites	567 tasks of 224 workstations in 18 plants	RULA > REBA	-3 risk levels -REBA has the ability to capture very awkward postures
Enez and Nalbantoğlu [22]	Timber harvesting in forestry	3119 postures of 58 workers	REBA > OWAS	4 risk levels
Kee [23]	Experimental environment	48 experimental postures	RULA > REBA > OWAS	4 risk levels
Kee and Karwowski [24]	Iron and steel, electronics, automotive and chemical industries, general hospital	301 postures	RULA > REBA > OWAS	The postures were classified and compared by industry, work type, and leg posture
Kee et al. [25]	Experimental environment	72 experimental postures	RULA > REBA = OWAS	-4 risk levels -Risk levels by OWAS and REBA were not significantly different
Domingo et al. [29]	Construction	14 postures	RULA > REBA	
Kee [30]	Automotive and its parts manufacturing industry, construction	209 postures	RULA > REBA > OWAS	4 risk levels
Pal and Dhara [36]	Uprooting job of rice cultivation	2 postures of 112 women cultivators	RULA = REBA > OWAS	
Isler et al. [37]	Clothing sector	4251 postures for REBA4237 postures for OWAS	REBA = OWAS	-No significant differences
Cremasco et al. [38]	Manual feeding of wood-chipper in forestry	7 tasks	RULA > REBA	Based on normalized values for RULA grand and REBA scores
Mukhopadhyay et al. [39]	Bicycle repairing	9 activities	RULA = REBA = OWAS	-All activities were assessed as the highest postural loads (action category/level: 4) -OWAS was used but based on different coding system
Balaji and Alphin [40]	Industrial vehicle driver cabin	Postures of 30 operators	RULA = REBA	-4 risk levels -No significant differences
Bhatia and Singla [41]	Kitchen	Postures of 30 participants	RULA = REBA	-No significant differences
Kulkarni and Devalkar [42]	5 activities in construction	30 workers	REBA > RULA	RULA assessed the activities as action level 3 or 4, and REBA as action level 4
Sain and Meena [43]	Clay brick kiln work	Postures of 154 workers	REBA > RULA	4 tasks: spading, mold filling, mold evacuating, brick carrying
Jones and Kumar [44]	Sawmill facility	15 saw-filers	RULA > REBA	3 risk levels
Jones and Kumar [45]	Sawmill facility	29 workers in four facilities	RULA > REBA	
Jones and Kumar [46]	Sawmill facility	87 sawmill workers	RULA > REBA	3 risk levels
Gallo and Mazzetto [47]	Forestry	18 frames	REBA > OWAS	
Garcia et al. [48]	Dental students	283 procedures of 75 students	RULA > OWAS	
Noh and Roh [49]	Dental hygienist	5 simulated working postures of three dental hygienists	RULA > REBA	
Qutubuddin et al. [50]	Saw mill	110 workers	RULA > REBA	
Qutubuddin et al. [51]	Automotive coach manufacturing	38 workers	RULA > REBA	
Sahu et al. [52]	Potter and sculptor	10 working postures of 80 male potters’ and 50 clay sculptors	RULA > REBA	
Shanahan et al. [53]	Rodworking in construction	25 tasks	RULA > REBA	
Ansari and Sheikh [54]	Small scale industry of India	15 workers	RULA > REBA	
Mukhopadhyay and Khan [55]	Meat cutters	8 tasks	RULA > REBA	OWAS was used but based on different coding system
Hussain et al. [56]	Furniture assembly	705–706 postures of 12 participants	REBA > OWAS	705 postures were used for REBA analysis and 706 postures for OWAS analysis
Chowdhury et al. [57]	Computer workstation	72 postures	RULA > REBA	
Ünver-Okan et al. [58]	Forest nurseries	10 works of 175 nurseries	RULA > REBA > OWAS	3 risk levels
Upasana and Vinay [59]	Tailors	60 male tailors in 14 boutique shops	RULA > REBA	
Boulila et al. [60]	Mechanical manufacturing	3 operators’ postures	RULA > REBA	
Dev et al. [61]	Welders	5 postures	RULA > REBA	
Landekić et al. [62]	Forest thinning	248 postures for 3 machines: chainsaw, forwarder and harvester	REBA > OWAS	4 risk levels
Li et al. [63]	Lifting tasks	13–18 postures according to 3 participants	RULA > REBA	
Joshi et al. [64]	Roof stick bending of public transport buses	7 processes	REBA > OWAS	
Kalkis et al. [65]	Metal processing	21 postures	RULA > REBA	
Khan and Deb [66]	Vendors selling edible items	8 vendors’ postures	RULA > REBA	
Paini et al. [67]	Wood harvesting	3 postures of 6 operators in tree cutting operations	RULA > REBA	
Vahdatpour and Sayed-Mirramazani [68]	Gastroenterologists	18 postures	RULA > OWAS	
Yayli and Çalişkan [69]	Forest nursery	104 forest nursery workers	RULA > REBA > OWAS	Based on hazardous ratios in working postures
Ijaz et al. [70]	Brick industry	Postures of 8 activities	RULA > REBA	
Kamath et al. [71]	Mechanical engineering laboratory	5 postures	RULA > REBA	
Qureshi and Solomon [72]	Foundry units	210 postures	RULA > REBA	

**Table 3:** Agreements between methods (%).

	OWAS and RULA	OWAS and REBA	RULA and REBA
Chiasson et al. [12]	-	-	73.7 (567) *
Joshi and Deshpande [19] **	37.5 (20)	36.4 (19)	25.0 (44)
Enez and Nalbantoğlu [22]	-	29.1 (3119)	-
Kee [23]	16.7 (48)	8.3	33.3
Kee and Karwowski [24]	29.2 (301)	54.8	48.2
Kee et al. [25]	33.3 (72)	52.8	29.2
Kee [30]	17.7 (209)	35.9	41.1
Pal and Dhara [36]	50.0 (2)	50.0	100.0
Cremasco et al. [38]	-	-	85.7 (7)
Kulkarni and Devalkar [42]	-	-	66.7 (30)
Jones and Kumar [46]	-	-	66 (87)
Gallo and Mazzetto [47]	-	33.3 (18)	-
Garcia et al. [48]	0 *** (283)	-	-
Noh and Roh [49]	-	-	20.0 (5)
Sahu et al. [52]	-	-	60.0 (10)
Ünver-Okan et al. [58]	40.0 (10)	50.0	50.0
Paini et al. [67]	-	-	33.3 (3)
Qureshi and Solomon [72]	-	-	75.24 (105)
Mean (±standard deviation)	28.1 ± 15.9	39.0 ± 14.9	53.8 ± 23.9

* The values in parenthesis are sample size; **: agreements and sample sizes for the OWAS and RULA, OWAS and REBA, and RULA and REBA are averaged values for 3, 4 and 5 studies; ***: ĸ value, respectively.

**Table 4:** Correlation coefficients between methods.

	OWAS and RULA	OWAS and REBA	RULA and REBA
Chiasson et al. [12]	-	-	0.67 *
Kee [23]	0.482 **	0.435 **	0.415 **
Kee and Karwowski [24]	0.511 *	0.487 **	0.468 **
Kee et al. [25]	0.491 **	0.785 **	0.691 **
Kee [30]	0.562 **	0.451 **	0.445 *
Mean (±standard deviation)	0.51 ± 0.04	0.54 ± 0.17	0.54 ± 0.13

*: significant at α = 0.05; **: significant at α = 0.05; -: not available.

**Table 5:** Intra- and inter-rater reliabilities by studies.

Methods	Study	Applied Fields	No. of Raters	Intra-Rater Reliability	Inter-Rater Reliability
OWAS	Karhu et al. [10]	Steel industry	4	70–100%	23–88% for workers A and B; 74–99% for work-study engineer 1 and 2
	de Bruijin et al. [73]	Nurses	2	88–97% for 4 weeks’ interval; 83–100% for 3.5 months’ interval	87–89%
	Kivi and Mattila [74]	Building industry	2	-	-86% for the back; -94% for the arms; -85% for the leg; -94% for the force
	Mattila et al. [75]	Building construction	2	-	-97% for the back postures; -100% for the arm postures; -98% for the leg postures; -97% for the whole body
	Lins et al. [76]	Laboratory settings	3	-	-Over 98% (ĸ = 0.98) for whole body; -80–96% (ĸ = 0.85) for the upper body; -66–97% (ĸ = 0.85) for the legs
	Widyanti [84]	Tofu, military equipment manufacturing, automotive maintenance and service, cracker, and milk processing	50	-	-% agreement: 57.07% -ĸ value: 0.39
RULA	McAtamney and Corlett [8]	Keyboard operations, packing, sewing and brick sorting tasks	120	-	High consistency
	Dockrell et al. [77] *	Computer work environment	6	0.27–0.86 for the action levels; 0.47–0.84 for the grand scores	-0.54–0.72 for the action levels; -0.50–0.77 for the grand scores
	Laeser et al. [78]	Computer workstation	-	-	-Kendall’s W = 0.773; -r = 0.96 between the independent observers’ and the lead investigator’s scores
	Breen et al. [79]	Computer workstation	3	-	94.6%
	Oates et al. [80]	Computer work environment	1	-	Ebel r = 0.73
	Widyanti [84]	Tofu, military equipment manufacturing, automotive maintenance and service, cracker, and milk processing	50	-	-% agreement: 58.25% -ĸ value: 0.20
REBA	Hignett and McAtamney [9]	-	14	-	62–85% (omitting the upper arm category)
	Lamarão et al. [81]	Textile industry, libraries, offices and supermarkets	2	0.104–0.504 ** (15.09–69.81%)	0.126–0.454 ** (5.66–66.03%)
	Schwartz et al. [82]	Custodial tasks	9	0.925 *	0.54 **
	Jantowitz et al. [83] **	Hospital settings	2	-	-0.54 for the upper body; -0.66 for the trunk/lower extremity; -<0.4 for the distal extremity
	Widyanti [84]	Tofu, military equipment manufacturing, automotive maintenance and service, cracker, and milk processing	50	-	-% agreement: 50.14% -ĸ value: 0.26

*: interclass correlation coefficients; **: ĸ value.

**Table 6:** Validations by studies.

Method	Study	Applied Fields	Sample Size	References Compared	Results
OWAS	Choi et al. [21] & Kong et al. [26]	Agriculture	196 postures	-Subjective ergonomic expert’s evaluations	OWAS action category was in ‘moderate’ agreement with the experts’ assessments (ĸ = 0.538 and 0.501, respectively) *
	Kee [23]	Experimental conditions	48 experimental postures	-Discomfort	OWAS action category was not significantly correlated with discomfort (r = −0.151, *p* > 0.10), and % capable at shoulder (r = −0.289, *p* > 0.05), but was correlated with % capable at trunk (r = −0.395, *p* < 0.01)
	Kee et al. [25]	Experimental conditions	72 experimental postures	-Discomfort -MHT	OWAS action category was not significantly correlated with discomfort and MHT (r = 0.125 (*p* > 0.10) and r = −0.151 (*p* > 0.10), respectively)
	Burdorf et al. [28]	Concrete manufacturing	1009 observations of 114 workers	-Prevalence of back pain	Average time spent working with a bent and/or twisted position of the back observed by the OWAS contributed to the prevalence
	Kee [30]	Automotive and its parts’ manufacturing, and construction industries	209 MSDs cases	-Association with MSDs	The OWAS action category was not significantly associated with MSDs (*p* > 0.10)
	Vahdatpour and Say-ed Mirramazani [68]	Gastroenterologists	18 postures	-Prevalence of MSDs	OWAS action level was not associated with the incidence of MSDs
	Widyanti [84]	Tofu, military equipment manufacturing, automotive maintenance and service, cracker, and milk processing	51 raters or postures in each industry	-Ratings between 50 new raters and an ergonomics expert for OWAS, RULA and REBA	Significant correlations between the ratings of the new raters and those of the expert for the OWAS (r = 0.802, *p* < 0.01)
	Kayis and Kothiyal [85]	manual materials handling tasks in several manufacturing industries	25 tasks	-L5/S1 compressive forces -Borg scale	Majority of the results of risk assessments (80%) were in agreement with one another
	Olendorf and Drury [86]	Experimental conditions	168 postures of 12 participants	-Perceived exertion -Body part discomfort measures	OWAS action levels and perceived exertion scores were associated
	Hellig et al. [87]	Experimental conditions	25 postures of 17 participants	-Ratings of perceived exertion (RPE), -Muscle activity	OWAS action levels were statistically significantly correlated with the RPE and back muscle activity
	Hellig et al. [89]	Experimental conditions	16 postures of 24 participants	-Muscle activity	OWAS action category was statistically significantly correlated with muscle activity (Spearman correlation coefficients: 0.17–0.55)
	van der Beek et al. [90]	Scaffolding tasks	26 workers	-Revised NIOSH lifting equation -Lifting guidelines for the Dutch construction industry (Arbouw method) -Rapid appraisal of the NIOSH lifting equation (practitioner’s method)	Ranks for 3 distinct scaffolding tasks determined by the OWAS was different from those determined by the other methods
RULA	McAtamney and Corlett [8]	Experimental conditions (VDU-based data-entry task)	2 postures of 16 operators	-perceived pain, ache, and discomfort	RULA scores are sensitive to pain, ache, or discomfort
	Choi et al. [21] & Kong et al. [26]	Agriculture	196 postures	-Subjective ergonomic expert’s evaluations	RULA action level was in ‘good’ and ‘moderate’ agreement with the experts’ assessments, respectively (ĸ = 0.599 and 0.627, respectively) *
	Kee [23]	Experimental conditions	48 experimental postures determined by hand positions and external loads	-Discomfort	RULA grand score was significantly correlated with discomfort (r = 0.554, *p* < 0.01), and % capable at trunk (r = −0.591, *p* < 0.01), but not with % capable at shoulder (r = −0.242, *p* < 0.05)
	Kee et al. [25]	Experimental conditions	72 experimental postures	-Discomfort -MHT	RULA grand score was significantly correlated with discomfort and MHT (r = 0.599 (*p* < 0.01) and r = −0.649 (*p* < 0.01), respectively)
	Yazdanirad et al. [27]	Pharmaceutical and automotive and assembly industries	210 workers	-Prevalence of subjective upper extremity musculoskeletal symptoms	RULA action levels were associated with the prevalence of the upper extremity MSDs
	Domingo et al. [29]	Construction	14 postures	-Subjective MSD symptoms	RULA scores had a negligible relationship with upper limb MSDs
	Kee [30]	Automotive and its parts’ manufacturing, and construction industries	209 MSDs cases	-Association with MSDs	RULA grand score and action level were significantly associated with MSDs (*p* < 0.01)
	Massaccesi et al. [31]	Driving rubbish-collection and road-washing vehicles	77 drivers’ postures	-Self-reported pain, ache, and discomfort	RULA trunk and neck scores were associated with pain, aches, and discomforts
	Shuval and Donchin [33]	Software communication industry	84 workers	-Prevalence of subjective upper extremity musculoskeletal symptoms	RULA hand/wrist scores were associated with the prevalence of the upper extremity symptoms
	Vahdatpour and Say-ed Mirramazani [68]	Gastroenterologists	18 postures	-Prevalence of MSDs	RULA score had a direct relationship with MSDs of the neck, upper back and knees
	Breen et al. [79]	Computer use	337 postures of 69 children	-Discomfort	Higher mean RULA grand score was correlated with discomfort
	Widyanti [84]	Tofu, military equipment manufacturing, automotive maintenance and service, cracker, and milk processing	51 raters or postures in each industry	-Ratings between 50 new raters and an ergonomics expert for OWAS, RULA and REBA	Significant correlations between the ratings of the new raters and those of the expert for the RULA (r = 0.799, *p* < 0.01)
	Fountain [93]	Experimental conditions (typing task)	3 postures of 20 participants	-EMG -Discomfort -Job attitude scores	RULLA risk level had a significant effect on perceived discomfort
REBA	Choi et al. [21] & Kong et al. [26]	Agriculture	196 postures	-Subjective ergonomic expert’s evaluations	REBA action level was in ‘moderate’ agreement with the experts’ assessments (ĸ = 0.578 and 0.490, respectively) *
	Kee [23]	Experimental conditions	48 experimental postures	-Discomfort	REBA score was significantly correlated with discomfort (r = 0.379, *p* < 0.01), and % capable at trunk (r = −0.609, *p* < 0.01), but not with % capable at shoulder (r = −0.272, *p* > 0.05)
	Kee et al. [25]	Experimental conditions	72 experimental postures	-Discomfort -MHT	REBA score was significantly correlated with discomfort and MHT (r = 0.352 (*p* < 0.01) and r = −0.465 (*p* < 0.01), respectively)
	Domingo et al. [29]	Construction	14 postures	-Subjective MSD symptoms	REBA scores had a weak relationship with entire body MSDs
	Kee [30]	Automotive and its parts’ manufacturing, and construction industries	209 MSDs cases	-Association with MSDs	REBA action level was significantly associated with MSDs (*p* < 0.01)
	Rathore et al. [32]	Glass artware industry	250 workers	-Prevalence of subjective musculoskeletal disorders	REBA scores and the musculoskeletal symptoms for the different body regions were significantly correlated
	Widyanti [84]	Tofu, military equipment manufacturing, automotive maintenance and service, cracker, and milk processing	51 raters or postures in each industry	-Ratings between 50 new raters and an ergonomics expert for OWAS, RULA and REBA	Significant correlations between the ratings of the new raters and those of the expert for the REBA (r = 0.790, *p* < 0.01)

*: Based on the quadratic weighted ĸ analysis.

## Data Availability

Not applicable.
